# Cases of Dislocation of the Shoulder

**Published:** 1846-07

**Authors:** Frederick Hyde

**Affiliations:** Cortlandville, N. Y.


ART. IV.—*Cases of Dislocation of the Shoulder. By* FREDERICK HYDE, M. D., *Cortlandville, N. Y.*

DR. FLINT:—By reference to my notes upon luxations, I find during ten years, the period of my professional practice, that the whole number of the shoulder is fourteen. These occurred in patients between 25 and 75 years of age, and, with two exceptions, in vigorous habits. In eleven, the head of the humerus occupied the axillary space; in the remaining three, it was found beneath the pectoral muscles, below the clavicle. The period between displacement and reduction, varied from one to ten hours in thirteen of the cases, while in the other, or remaining one, it was thirty-five days. They were all reduced with little difficulty, and followed by restoration of function. The treatment was governed by the following rules. First, patients were placed upon a low seat, in an upright position. Second, a firm broad sheet, or something equivalent, was placed around the chest, making secure its ends at some distance from the patient, upon the sound side. Third, made immoveable the scapula. Fourth, applied, generally, extending band above the elbow. Fifth, in patients of great muscular power, always procured a general relaxation, previous to making the extension. Sixth, made extension in the direction in which the arm was found, or in whatever direction would provoke least, the antagonizing muscular power. Seventh, always applied extension power gradually, suspending, and making it stationary, at proper intervals, to encourage muscular fatigue. Eighth, taking the precaution, when using the knee, or something else, placed in the axilla, not to allow pressure upon either of its walls, made by the latissimus dorsi and pectoralis-major muscles. A history of the case referred to above, of thirty-five days standing, may not be amiss.

S. M., aged 55, of vigorous habit, resident of Virgil, Cortland Co., in the act of stepping into his house, late at night, fell, striking the palm of his right hand upon the threshold of the door, upon which he soon felt severe pain in the shoulder and arm, with numbness and insensibility of the limb. He remained in this condition until sometime next day, when his physician saw him. He said he found the shoulder and arm much swollen, painful and tender, that the patient could not move the arm a particle. He gave him thorough antiphlogistic treatment, and continued his attendance upon him between two and three weeks, when the inflammation had so much subsided that he dismissed him. Patient took charge of himself another week, and the arm continuing useless, he began to enquire whether there might not be a dislocation. Under these circumstances, he was brought to me for advice. I found the downward luxation, with the head of the bone, of course, resting firmly in the axilla. Said to him that it could probably be reduced with safety. Being able to travel, he spent several days more for the purpose of consulting different surgeons. He returned to me on the thirty-fourth day from the injury, said he was ready, and wished me to make the trial of reduction, which I did the next day, assisted by Dr. Lansing, as follows: Placed the patient upon a low firm seat, applied a strong sheet around the trunk, the distal ends of which were fastened to an immoveable fixture opposite to the injured shoulder. Secured scapula firmly to obviate motion. Applied extending band above elbow, and put it into the hands of two assistants. Next opened freely a vein in the sound arm, from which he soon become faint, when the extension was commenced, and increased, until the faintness was gone, then held stationary while the blood was again allowed to flow to faintness, upon which, the extending power was immediately renewed, and continued until the faintness had again subsided. The muscles evincing evident fatigue, some rotary motions were given to the arm. The bleeding was once more permitted to return to slight faintness, upon which the extension was again increased, while with my knee in the axilla, and carrying down a little the arm, the head of the bone was felt to move. The extension being kept at this point, an effort was made with the knee to raise a little the head of the humerus, at the same time lowering its opposite extremity, when it readily returned into the glenoid cavity. The ordinary dressing was applied. It was several weeks before he recovered a tolerable use of the limb, but eventually the recovery was complete. In reviewing the history of this case, one circumstance will be noticed which materially influenced the prognosis. I refer to the early adoption of energetic means to reduce the inflamation. There can be no doubt that it limited very much the adhesions, lessening the extent of lacerations in the reduction, and in particular the liability to rupture the axillary artery that exists in cases accompanied with the legitimate results of inflammation. Yours, &c.

CORTLANDVILLE, May 14, 1846. FREDERICK HYDE.

